# The effect of carbon dioxide on near-death experiences in out-of-hospital cardiac arrest survivors: a prospective observational study

**DOI:** 10.1186/cc8952

**Published:** 2010-04-08

**Authors:** Zalika Klemenc-Ketis, Janko Kersnik, Stefek Grmec

**Affiliations:** 1 Department of Family Medicine, Medical School, University of Maribor, Slomškov trg 15, 2000 Maribor, Slovenia; 2 Department of Family Medicine, Medical School, University of Ljubljana, Poljanski nasip 58, 1000 Ljubljana, Slovenia; 3 Faculty of Health Sciences, University of Maribor, Zitna ulica 15, 2000 Maribor, Slovenia; 4 Center for Emergency Medicine, Ljubljanska 5, 2000 Maribor, Slovenia

## Abstract

**Introduction:**

Near-death experiences (NDEs) are reported by 11-23% of cardiac arrest survivors. Several theories concerning the mechanisms of NDEs exist - including physical, psychological, and transcendental reasons - but so far none of these has satisfactorily explained this phenomenon. In this study, we investigated the effect of partial pressures of O_2 _and CO_2_, and serum levels of Na and K on the occurrence of NDEs in out-of-hospital cardiac arrest survivors.

**Methods:**

A prospective observational study was conducted in the three largest hospitals in Slovenia. Fifty-two consecutive patients (median age 53.1 years, 42 males) after out-of-hospital cardiac arrest were included. The presence of NDEs was assessed with a self-administered Greyson's NDE scale. The initial partial pressure of end-tidal CO_2_, the arterial blood partial pressures of O_2 _and CO_2 _and the levels of Na and K in venous blood were analysed and studied. Univariate analyses and multiple regression models were used.

**Results:**

NDEs were reported by 11 (21.2%) of the patients. Patients with higher initial partial pressures of end-tidal CO_2 _had significantly more NDEs (*P *< 0.01). Patients with higher arterial blood partial pressures of CO_2 _had significantly more NDEs (*P *= 0.041). Scores on a NDE scale were positively correlated with partial pressures of CO_2 _(*P *= 0.017) and with serum levels of potassium (*P *= 0.026). The logistic regression model for the presence of NDEs (*P *= 0.002) explained 46% of the variance and revealed higher partial pressures of CO_2 _to be an independent predictor of NDEs. The linear regression model for a higher score on the NDE scale (*P *= 0.001) explained 34% of the variance and revealed higher partial pressures of CO_2_, higher serum levels of K, and previous NDEs as independent predictors of the NDE score.

**Conclusions:**

Higher concentrations of CO_2 _proved significant, and higher serum levels of K might be important in the provoking of NDEs. Since these associations have not been reported before, our study adds novel information to the field of NDEs phenomena.

## Introduction

Near-death experiences (NDEs) are an unexplained but quite common experience in many cardiac arrest patients after successful resuscitation [1]. One definition describes NDEs as deep psychological experiences with feelings of transcendence or mystical encounter that typically occur in persons close to death or in situations of intense physical or emotional danger [2]. These elements may include cognitive components such as accelerated thought processes and a 'life review', affective components such as peacefulness and joy, or transcendental components such as apparent encounters with mystical entities or deceased persons [2].

Although several theories explaining the mechanisms of NDEs exist, so far none of them have completely explained the phenomenon. Physiological theories regard NDEs as a part of the physiological processes that accompany the act of dying [3]. The factors that could be important in provoking NDEs are anoxia [4-7], hypercapnia [3,5], and the presence of endorphins [5,8], ketamine [9], and serotonin [10], or abnormal activity of the temporal lobus [7,11-15] or the limbic system [16,17]. These psychological theories try to explain the NDEs as a way of dissociation [18], depersonalisation [19,20], reactivation of birth memories [21], and regression [22,23]. Transcendental theories regard NDEs as unambiguous proof for the existence of life after death and the existence of the soul (or spirit) as a separate entity [1,5,24].

Few prospective studies reported an incidence of NDEs of 11 to 23% in cardiac arrest survivors [3,25-27]. Younger patients seem to experience NDEs more often [18,25,28]. Also, a higher serum partial pressure of oxygen (pO_2_) has been shown to be associated with the occurrence of NDEs [3]. Other factors that might be important are the cardiac aetiology of cardiac arrest [27], previous near-death or paranormal experiences [27], out-of-hospital cardiac arrest [25], female sex [25], and fear of death [25].

The aim of this study was to investigate the effect of serum pO_2_, serum partial pressure of carbon dioxide (pCO_2_), and partial pressure of end-tidal carbon dioxide (petCO_2_) on the occurrence of NDEs in out-of-hospital cardiac arrest survivors. In addition, we also investigated the effect of serum levels of sodium and potassium on the occurrence of NDEs.

## Materials and methods

### Study population

We studied out-of-hospital cardiac arrest survivors who were successfully resuscitated in out-of-hospital settings and consecutively admitted to intensive care units from the beginning of January 2008 to the end of June 2009. The inclusion criteria were: 18 years old or older, presence of the cardiac aetiology of cardiac arrest (as confirmed during the resuscitation and later hospital work up), clinical death (defined as a cessation of breathing and effective cardiac output - electrocardiogram patterns of ventricular fibrillation, pulseless ventricular tachycardia, pulseless electrical activity, and asystolia detected by pre-hospital resuscitation teams), a post-resuscitation cerebral performance categories scale score of 1 [29], and the patients' informed consent.

The National Medical Ethics Committee approved the study - No. 79/10/07.

### Settings

We conducted a multicentre study in the intensive care units of three of the largest hospitals in Slovenia: the Clinical Centre of Ljubljana, the Clinical Centre of Maribor, and the General Hospital of Celje. The majority of cardiac arrest survivors in Slovenia are transferred to these three hospitals. At the same time, each of these hospitals is closely connected to several regional out-patient emergency medical centres.

Regional out-patient emergency medical centres are part of primary care out-patient healthcare centres. Teams of two medically trained paramedics and one emergency physician provide urgent medical care for the population of their catchment areas. Critically ill patients are transferred to the nearest regional hospital.

### Data collection

**Figure 1:**
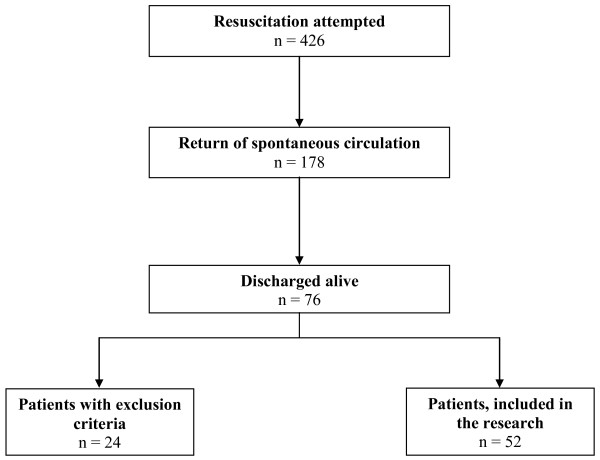
**The flowchart of patients' recruitment**. The flowchart starts with the number of out-of-hospital cardiac arrest patients, in whom resuscitation was attempted, followed by the number of patients with return of spontaneous circulation, then the number of patients discharged from the hospital alive, and finally the number of patients that were included in the study.

Eligible patients were approached during their hospital stay by a member of the research team, who explained the purpose of the study, assured their complete anonymity, and obtained their informed consent (Figure 1). No patients refused the interview. Then they filled in a self-administered questionnaire about the NDEs [see Additional file 1] [20], which consists of 16 questions about the cognitive, affective, paranormal, and transcendental component of NDEs. The questions could be answered on a three-point scale (from 0 to 2), with a minimum score of 0 and a maximum of 32. The total number of scores of 7 or above defines the existence of a NDE. The questionnaire was translated from English to Slovene using the guidelines recommended by Guillemin and co-workers [30]. Other data obtained with the interview with the patients were: sex, age, level of education, religious belief, previous NDEs, and fear of death before and after the cardiac arrest (Table 1).

**Table 1:** Patients' characteristics

Characteristic	Number (%) of patients	Number (%) of patients with NDEs
Sex		
Male	42 (80.0)	10 (23.8)
Female	10 (19.2)	1 (10.0)
Age		
<60 years old	35 (67.3)	6 (29.4)
≥ 60 years old	17 (32.7)	5 (17.1)
Education		
Primary	10 (19.2)	2 (20.0)
Vocational	20 (38.5)	2 (10.0)
Secondary	14 (26.9)	3 (21.4)
University	8 (15.4)	4 (50)
Religious belief		
Catholic	27 (51.9)	3 (11.1)
Muslim	4 (7.7)	1 (25.0)
Atheist	21 (40.4)	7 (33.3)
Fear of death before cardiac arrest		
Yes	10 (19.2)	2 (20.0)
No	42 (80.8)	9 (21.4)
Fear of death after cardiac arrest		
Yes	10 (19.2)	2 (20.0)
No	42 (80.8)	9 (21.4)
Previous NDEs		
Yes	2 (3.8)	2 (100.0)
No	50 (96.2)	9 (18.0)

NDE, near-death experience.

Data obtained from patients' files were: time until the beginning of resuscitation, time until return of spontaneous circulation (ROSC), drugs received during resuscitation, the initial petCO_2 _(in kPa), pO_2 _and pCO_2 _(both in kPa) in peripheral arterial blood, and serum levels of sodium and potassium (in mmol/l) in peripheral venous blood. Only the blood sample analysis that was performed on the samples taken in the first five minutes upon the admission of the patients to the hospital was taken into account.

### Statistical analysis

To analyse the data, we used the statistical package for the social sciences, version 13.0 (SPSS Inc, Chicago, IL, USA). The limit of statistical significance was set at *P *< 0.05. Descriptive statistics were computed. For the questionnaire, we calculated the reliability coefficient, Cronbach α. Patients with a NDE score of 7 or above were assigned to the NDE group, others were assigned to the non-NDE group [20]. To identify statistically significant differences between different variables, we used an independent samples t-test, chi-squared test, and a Wilcoxon rank sum test. Linear correlation analysis was performed to reveal possible correlations. To identify a possible model for the explanation of differences, linear and binary logistic regressions were performed. The variables that showed statistically significant differences in univariate analysis were entered into multivariate analysis.

## Results

### Descriptive data

The study included 52 patients (Figure 1). NDEs were reported by 11 (21.2%) of them (Table 1). The mean (standard deviation) NDE score of all patients was 3.2 ± 5.0 points. The average NDE score of patients in the NDE group was 11.5 ± 4.4, and of the non-NDE group was 0.9 ± 1.6. The Cronbach α of the questionnaire was 0.875. The average age of the patients was 53.1 ± 14.5 years. The average time until the beginning of resuscitation was 4.2 ± 3.7 minutes. The average time until ROSC was 8.7 ± 5.6 minutes. During the resuscitation, 39 (75.0%) patients received drugs. Epinephrine was given to 27 (51.9%), amiodarone to 16 (30.8%), atropine to 13 (25.0%), vasopressin to 9 (17.3%), sodium bicarbonate to 5 (9.6%), lidocaine and magnesium sulphate to 3 (5.8%), and erythropoietin and calcium gluconate to 1 (1.9%) patients. The average petCO_2 _was 5.1 ± 1.2 kPa. The average pO_2 _was 23.3 ± 14.6 kPa and pCO_2 _was 5.6 ± 1.6 kPa. The average serum level of sodium was 140.1 ± 4.5 mmol/l and potassium was 4.2 ± 0.9 mmol/l.

### Univariate analysis

**Figure 2:**
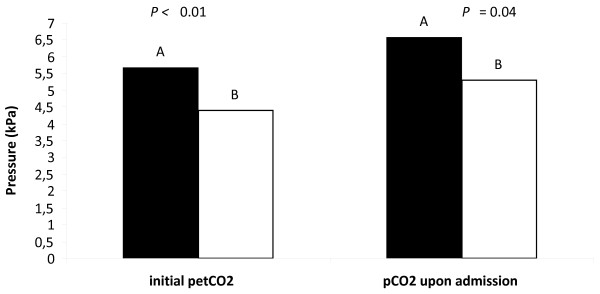
**Differences in pCO_2 _among near-death experience and non-near-death experience groups**. The graph presents the statistically significant differences in initial partial pressure of end-tidal carbon dioxide (petCO_2_) and partial pressure of carbon dioxide (pCO_2_) in arterial blood upon admission to hospital (assessed in the first five minutes upon admission). A, near-death experience group; B, non-near-death experiences group.

Patients with higher petCO_2 _had significantly more NDEs (5.7 ± 1.1 vs. 4.4 ± 1.2, *P *< 0.01; Table 2 and Figure 2). Patients with higher pCO_2 _had significantly more NDEs (6.6 ± 2.3 vs. 5.3 ± 1.4, *P *= 0.041; Table 2). Patients with previous NDEs had significantly more NDEs (100% vs. 18.0%, chi squared = 7.753, *P *= 0.041). The NDE score was positively correlated with pCO_2 _(r = 0.366, *P *= 0.017) and with the serum level of potassium (r = 0.315, *P *= 0.026). Patients with lower pO_2 _had more NDEs, although the difference was not statistically significant (16.4 ± 11.1 vs. 25.3 ± 15.0, *P *= 0.108). The occurrence of NDEs did not correlate with the patients' sex, age, level of education, religious belief, fear of death, time to ROSC, drugs during resuscitation, or serum sodium levels (Table 2).

**Table 2:** Correlation of independent variables with the presence of NDEs

Variable	NDEs group (mean ± SD)	Non-NDEs group (mean ± SD)	*P*
Age (years)	57.9 ± 13.8	51.8 ± 14.6	0.217
Time until ROSC (minutes)	8.3 ± 6.7	8.8 ± 5.3	0.772
petCO_2 _(kPa)	5.7 ± 1.1	4.4 ± 1.2	< 0.01
pO_2 _(kPa)	16.4 ± 11.1	25.3 ± 15.1	0.108
pCO_2 _(kPa)	6.6 ± 2.3	5.3 ± 1.4	0.041
Serum sodium (mmol/l)	139.2 ± 6.1	140.4 ± 4.0	0.439
Serum potassium (mmol/l)	4.6 ± 1.2	4.1 ± 0.8	0.118

NDE, near-death experience; petCO_2_, initial partial end-tidal pressure of carbon dioxide; pCO_2_, partial pressure of carbon dioxide; pO_2_, partial pressure of oxygen; ROSC, return of spontaneous circulation; SD, standard deviation.

### Multivariate analysis

Higher pCO_2 _was an independent predictor of NDEs. The logistic regression model explained 46% of the variation (Table 3). A higher NDE score was independently associated with higher pCO_2_, higher serum levels of potassium, and previous NDEs. The linear regression model explained 34% of the variation (Table 4).

**Table 3:** Logistic regression model for the presence of NDEs

Variable	Odds ratio (e^B^)	Lower CI^†^	Upper CI	*P*
Previous NDEs	2E+010	0		0.999
pCO_2 _(kPa)	1.917	1.120	3.282	0.018
Potassium (mmol/l)	1.947	0.820	4.628	0.131
Constant	0			0.006

Chi-squared = 14.838, df = 3, *P *= 0.002.

CI, confidence interval; NDE, near-death experiences; pCO_2_, partial pressure of carbon dioxide.

**Table 4:** Linear regression model for the higher NDE score

Variable	B	Lower CI	Higher CI	*P*
Previous NDEs	6.529	0.400	12.658	0.037
pCO_2 _(kPa)	1.165	0.362	1.968	0.006
Potassium (mmol/l)	1.659	0.299	3.019	0.018
Constant	-10.598	-17.870	-3.327	0.005

Sum of squares = 331.263, df = 3, *P *= 0.001.

CI, confidence interval; NDE, near-death experiences; pCO_2_, partial pressure of carbon dioxide.

## Discussion

Our prospective study reports a 21.2% incidence of NDEs in out-of-hospital cardiac arrest survivors. It also suggests that the occurrence of NDEs is connected to higher initial petCO_2_, higher arterial blood pCO_2_, and previous NDEs. Higher serum levels of potassium might also play a role.

To our knowledge, this is the first prospective study to report a possible correlation between NDEs and CO_2_. It is still not clear whether NDEs occur before, during, or after the period of cardiac arrest [3]. During cardiac arrest, the petCO_2 _falls to very low levels, reflecting the very low cardiac output achieved with cardiopulmonary resuscitation [31]. Higher levels of petCO_2 _therefore indicate better cardiac output and higher coronary perfusion pressure [32]. Our findings concerning the association between initial petCO_2 _and the occurrence of NDEs therefore support the hypothesis that NDEs occur during the cardiac arrest.

On the other hand, the association between higher pCO_2 _upon admission and the occurrence of NDEs might suggest that NDEs occurs after the cardiac arrest. But higher pCO_2 _upon admission might simply reflect higher initial petCO_2_. Nevertheless, it is known that CO_2 _changes the acid-base equilibrium in the brain, which can provoke unusual experiences in the form of bright light, visions, and out-of-body or even mystical experiences [3,5]. Some earlier studies have shown that inhaled CO_2_, used as a psychotherapeutic agent, could cause NDE-like experiences [33,34]. Therefore, we can conclude that CO_2 _might be one of the major factors for provoking NDEs, regardless of when NDEs occur. As far as we know, serum levels of potassium were assessed only in one study [3]. The mean level of potassium in the NDE group was slightly lower in comparison to the control group, but no significant differences were found. As our study managed to associate serum levels of potassium only with the higher NDE score, and not also with the higher incidence of NDEs, no firm conclusions can be drawn at this point. Also, the possible mechanism of the effect of potassium in the NDEs has not yet been established. Alternative theories found the explanation for NDEs in quantum theory, which suggests that consciousness may arise from quantum processes within neuronal microtubules [35]. The recent work of Bernroider and Roy suggests that quantum entanglement in the ion channels (especially in the potassium channel) of brain cells underlies information processing in the brain and, ultimately, also consciousness [36]. Although untenable and purely theoretic, this possible connection between potassium channels in the brain and the mechanism of consciousness (and therefore the possible mechanism of NDEs) deserves further investigation.

Available data on the role of oxygen in provoking NDEs is ambiguous. Although one physiological theory [5] suggests that anoxia (or hypoxia) might be the cause for NDEs, Parnia and colleagues [3] found a higher mean pO_2 _in peripheral blood; however, due to an insufficient sample quantity, a univariate analysis was not performed. In our study, the NDE group had a lower mean pO_2 _than the non-NDE group, but this difference was not statistically significant (Table 2). Nevertheless, this finding is in favour of the theory of anoxia [5] and supported by several studies that reported NDE-like experiences in decreased cerebral perfusion (resulting in local cerebral hypoxia) in rapid acceleration during training of fighter pilots [37], in hyperventilation followed by the valsalva maneuver [38], and in people exposed to high altitudes [6]. The proposed mechanism is the induction of hyperactivity of *N*-methyl *D*-aspartate (NMDA) receptors by hypoxia, which induces hallucination and might induce NDEs [10].

Previous prospective studies on NDEs reported an 11 to 23% incidence between cardiac arrest survivors [3,25-27], which is consistent with the incidence found in our study. We have not demonstrated the connection between younger age and a higher incidence of NDEs. In fact, the mean age of the NDE group was lower than the non-NDE group, but this difference was not statistically significant. Previous studies have shown that NDEs more often occur in patients younger than 60 years of age [3,27,28]. The age difference in our study might be overlooked due to an insufficient number of subjects. It is also true that almost 70% of patients in our sample were younger than 60 years. The mean age of patients in our sample was lower (for almost 10 years) than in the two largest prospective studies of NDEs in cardiac arrest survivors [25,27]. This difference might also be the reason why we were not able to demonstrate any age differences in the occurrence of NDEs.

Our study confirmed the findings of other studies on NDEs that sex [25,27], level of education [25,28], fear of death [25], time until ROSC [25,28], medication during resuscitation [25,28], serum level of sodium [3], and religious belief [25] are not associated with NDE occurance. It also confirmed previously reported findings [25] that patients with previous NDEs are more likely to have repeated NDEs in case of a new cardiac arrest episode.

The questionnaire proved to be a reliable instrument for assessing NDEs also in Slovenian. The Cronbach's α of the questionnaire in the original study was 0.88 [20] and our result (0.875) was almost the same.

Our study suggests that some physiological factors or processes might be important in provoking NDEs. On the other hand, the experiences induced by neurophysiological processes mostly consist of fragmented and random memories and confused experiences unlike the real NDEs that are clear, highly structured and easily recalled [3,25]. It is not thought possible to explain NDEs only in terms of physiological processes. Most likely multiple physiological factors are involved [5]. Clearly, the presence of NDEs pushes the current knowledge of human consciousness and mind-brain relation to the edge of our understanding.

The main strength of our study is its prospective design. With a consecutive recruitment of the patients and the inclusion of three of the largest Slovenian hospitals, the selection bias was reduced as much as possible. The use of a standardised scale for NDEs ensured the consistency of NDEs reports. The number of patients in the sample is the main weakness of our study. Therefore, some important differences might have been overlooked and the results should be interpreted with care. Also, receiver-operator characteristic curves for defining a threshold CO_2 _were not produced due to too small a number of patients. The weakness is also the fact that almost 70% of the patients in a sample were younger than 60 years old, which could affect the incidence and the demonstration of age differences in NDEs.

Further multicentre studies should investigate the effect of CO_2 _and potassium on the incidence of NDEs in a larger prospective sample of cardiac arrest patients or unconscious patients. The clinical reliability and relevance of our findings should be extensively studied.

## Conclusions

As much as one-fifth of out-of-hospital cardiac arrest patients report NDEs during cardiac arrest. Higher initial petCO_2 _and higher arterial blood pCO_2 _proved to be important in the provoking of NDEs. Higher serum levels of potassium might also be important. As these associations have not been reported before, our study adds new and important information to the field of NDE phenomena. As quality of life of NDE patients might be affected, NDEs warrant further study. Likewise, more rigorous measures to establish good acid-base equilibrium should be adopted in resuscitation guidelines.

## Key messages

• The incidence of NDEs in out-of-hospital cardiac arrest survivors is 21.2%.

• NDE occur more often in patients with higher petCO_2 _and pCO_2_.

• Higher serum levels of potassium correlate with higher score on Greyson's NDE scale.

• NDEs occur more often in patients with previous NDEs.

## Abbreviations

NDE: near-death experience; ROSC: return of spontaneous circulation; petCO_2_: partial pressure of end-tidal carbon dioxide; pCO_2_: partial pressure of carbon dioxide; pO_2_: partial pressure of oxygen; NMDA receptors: *N*-methyl *D*-aspartate receptors.

## Competing interests

The authors declare that they have no competing interests.

## Authors' contributions

ZKK was involved in the writing of the study protocol, ran the interviews with the patients, collected the data, analysed and interpreted the data, and wrote the first and second drafts of the manuscript. JK was involved in the designing of the study protocol, supervised the study, interpreted the data, and made comments to the first and second drafts of the manuscript. SG was involved in the designing of the study protocol, interpreted the data, and made comments to the first and second drafts of the manuscript.

## Supplementary Material

Additional file 1 The near-death experience scale. Click here for file
