# Supplementary material for: The effect of carbon dioxide on near-death experiences in out-of-hospital cardiac arrest survivors: a prospective observational study
Source: Crit Care. 2010 Apr 8;14(2):R56. doi: 10.1186/cc8952 (PMC2887177; doi:10.1186/cc8952)
Supplement: Additional file 1 — The near-death experience scale. [file cc8952-S1.DOC]

Appendix: The NDE scale

1. Did time seem to speed up?

2 – Everything seemed to be happening all at once

1 – Time seemed to go faster than usual

0 – Neither

2. Were your thoughts speeded up?

2 – Incredibly fast

1 – faster than usual

0 – Neither

3. Did scenes from your past come back to you?

2 – Past flashed before me, out of my control

1 – Remembered many past events

0 – Neither

4. Did you suddenly seem to understand everything?

2 – About the universe

1 – About myself or others

0 – Neither

5. Did you have a feeling of peace or pleasantness?

2 – Incredible peace or pleasantness

1 – Relief or calmness

0 - Neither

6. Did you have a feeling of joy?

2 – Incredible joy

1 – Happiness

0 – Neither

7. Did you feel a sense of harmony or unity with the universe?

2 – United, one with the world

1 – No longer in conflict with nature

0 – Neither

8. Did you see or feel surrounded by a brilliant light?

2 – Light clearly of mystical or other-worldly origin

1 – Unusually bright light

0 – Neither

9. Were your senses more vivid than usual?

2 – Incredibly more so

1 – More than usual

0 – Neither

10. Did you seem to be aware of things going on elsewhere, as if by ESP?

2 – Yes, and facts later corroborated

1 – Yes, but facts not yet corroborated

0 – Neither

11. Did scenes from the future come to you?

2 – From the world’s future

1 – From personal future

0 – Neither

12. Did you feel separated from your physical body?

2 – Clearly left the body and existed outside it

1 – Lost awareness of the body

0 – Neither

13. Did you seem to enter some other, unearthly world?

2 – Clearly mystical or unearthly realm

1 – Unfamiliar, strange place

0 – Neither

14. Did you seem to encounter a mystical being or presence?

2 – Definite being, or voice clearly of mystical or other-worldly origin

1 – Unidentifiable voice

0 – Neither

15. Did you see deceased spirits or religious figures?

2 – Saw them

1 – Sensed their presence

0 – Neither

16. Did you come to a border or point of no return?

2 – A barrier I was not permitted to cross; or “sent back” to life involuntarily

1 – A conscious decision to “return” to life

0 – Neither
